# Progressive acetabular dysplasia in a boy with mucopolysaccharoidosis type IV A (Morquio syndrome): a case report

**DOI:** 10.1186/1757-1626-1-410

**Published:** 2008-12-22

**Authors:** Ali Al Kaissi, Klaus Klaushofer, Franz Grill

**Affiliations:** 1 Ludwig Boltzmann Institute of Osteology, at the Hanusch Hospital of WGKK and, AUVA Trauma Centre Meidling, 4th Medical Department, Hanusch Hospital, Vienna, Austria; 2 Orthopaedic Hospital of Speising, Paediatric Department, Vienna, Austria

## Abstract

**Background:**

Morquio syndrome is an autosomal recessive lysosomal storage disorder, a mucopolysaccharidosis (PMS), characterized by abnormal metabolism of glycosaminoglycans. Major treatable concerns in patients with MPS type IV involve C1 to C2 instability, genu valgum, and hip subluxation. All patients demonstrate characteristic acetabular dysplasia and failure of ossification of the superolateral femoral head.

**Case presentation:**

We report on a 6-year-old boy whose prime presentation was a waddling gait associated with pain since early childhood. Radiographic documentation showed progressive acetabulo-femoral dysplasia associated with additional skeletal deformities. Laboratory investigations showed increased urinary keratan sulfate and reduced leukocyte enzymatic activity of N-Acetyl-Galaktosamin-6-sulfate-sulfatase. Mucopolysaccharoidosis type IV A (Morquio syndrome) has been identified.

**Conclusion:**

Patients with Morquio syndrome usually appear normal at birth but exhibit growth failure and spondyloepiphyseal dysplasia as infants. Most children are brought to a physician for investigation of what the parents perceive as an abnormal appearance by 12 to 18 months of age and thoracic kyphosis is supposed to be the first appearing deformity. In our present patient progressive acetabular dysplasia was the prime orthopaedic presentation causing effectively the development of a painful waddling gait.

## Background

Morquio [1] in Montevideo, Uruguay, and Brailsford [2] in Birmingham, England, simultaneously and independently described the entity now known to result from a deficiency of galactosamine-6-sulfatase. Morquio [1] observed the disorder in 4 sibs in a family of Swedish extraction. Notable features included osseous dystrophy, corneal clouding, aortic valve disease, and urinary excretion of keratosulfate. Clinically the condition is characterised by severe skeletal abnormalities with cloudy corneae and aortic regurgitation. Onset may be in the first two years of life with genu valgum, a short trunk and neck, pectus carinatum and coarse facies. Clouding of the cornea is mild but deafness may be a problem. The joints may be loose without the claw hand seen in other mucopolysaccharidoses. Odontoid hypoplasia leads to atlantoaxial instability. Although the skeletal changes may result in neurologic complications, but patients with the severe phenotype usually do not survive past the second or third decade of life. In type A Morquio there is deficiency of the enzyme galactosamine-6-sulphatase causing faulty degradation of keratan sulphate with glycosaminoglycan deposits in the body tissues. Keratan sulphate is excreted in large amounts in urine. In type B Morquio syndrome there is deficiency of the enzyme beta galactosidase. The phenotype however is milder than in type A [1-4].

## Clinical report

At the age of six-years the boy was referred to the orthopaedic department because of severe limping, waddling gait associated with hip pain. He was a product of a 38-weeks of uneventful gestation. At birth his growth parameters were around the 50 th percentile. Growth retardation has been noticed after the second year of life. Parents were healthy and unrelated (though from the same geographical area). Family history was non-contributory. The parents have observed no specific abnormality in his first two years of life. At his third-fourth year a waddling gait with frequent falls was the main abnormality. Duchenne muscular dystrophy was suspected in another institute. The latter was followed by a series of neuromuscular investigations that were proved normal.

**Figure 1:**
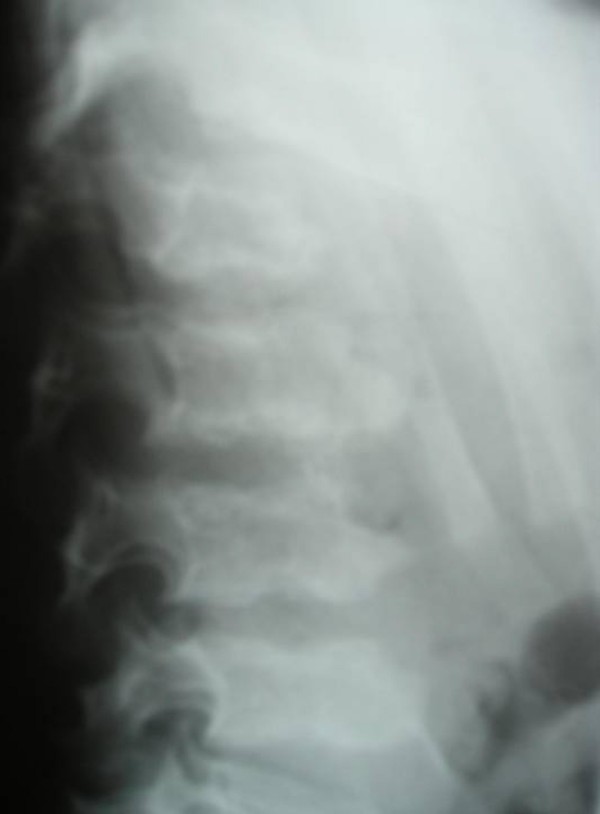
**Lateral spine radiograph showed significant platyspondyly**. A central tongue/anterior beaking associated with narrow discs was evident.

**Figure 2:**
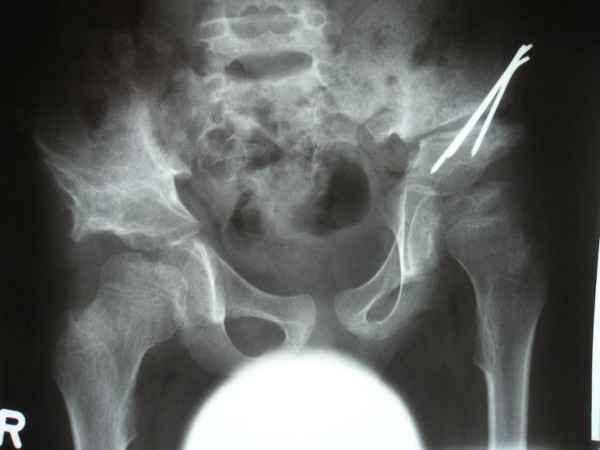
**Anteroposterior pelvis radiograph showed irregular ossification of the epiphyses**. Defective ossification of the femoral heads (significant flattening of the capital femoral epiphyses associated with widened femoral necks, dysplastic acetabulae and coxa valga. At this age (six years) a varus derotation osteotomy and a Salter osteotomy was performed.

**Figure 3:**
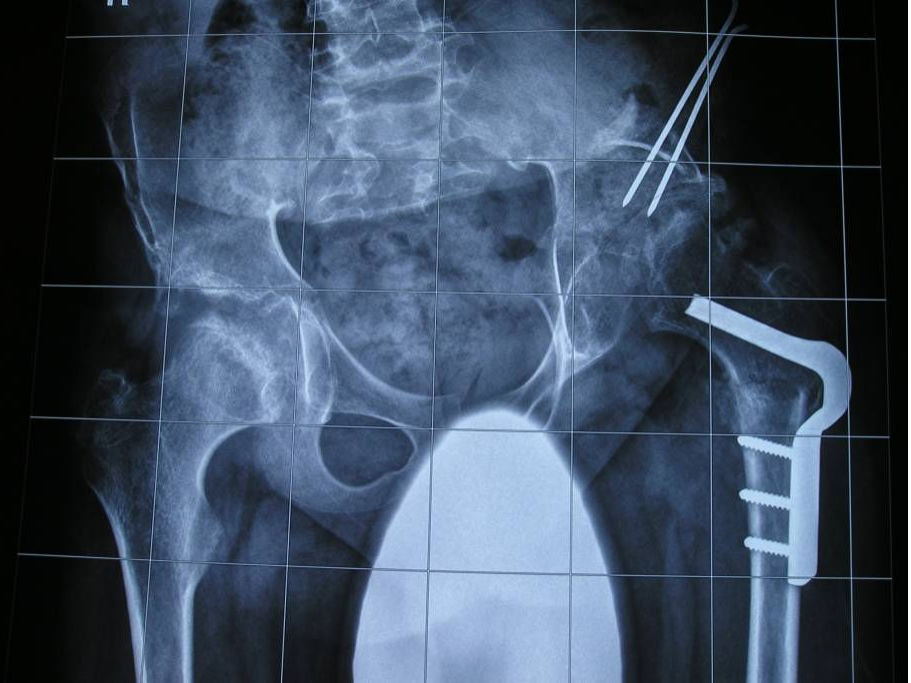
**Anteroposterior pelvis radiograph showed progressive hip deformity in connection with progressive dislocation and deficiency of the acetabulum**. There was posterior dislocation of the left hip at the age of 11 years. Proximal femoral osteotomy and a redirectional osteotomy of the acetabulum have been performed accordingly. During the following years the right hip developed nicely and was stable.

Clinical examination at the age of six-years showed severe growth deficiency of -3SD. Craniofacially, deep-seated eyes, depressed nasal bridge, and a prominent maxilla, however, no coarse facial features have been encountered. Relative ligamentous hyperlaxity was noted. Thoracolumbar kyphosis associated with short trunk dwarfism and a pigeon chest was evident. The hips were flexed in a crouched position and the head thrust forward and sunk between the shoulders. Shortness of the hands and feet was evident. The combined abnormalities had resulted in a duck-waddling gait. The radiographic features were distinctive. The vertebral bodies in the thoracic and the lumbar spine were platyspondylic. A central tongue/anterior beaking associated with narrow discs was evident (fig 1). Irregular ossification of the epiphyses was generalised. Defective ossification of the femoral heads (significant flattening of the capital femoral epiphyses associated with widened femoral necks, dysplastic acetabulae and coxa valga. At this age (six years) a varus derotation osteotomy and a Salter osteotomy was performed (fig 2). In this patient malalignment, instability and unfavourable anatomy were the main surgical problems. The hip deformity created problems because of progressive dislocation and deficiency of the acetabulum, despite, the fact that the right hip developed nicely and was stable. There was posterior dislocation of the left hip at the age of 11 years. Proximal femoral osteotomy and a redirectional osteotomy of the acetabulum have been performed accordingly (fig 3). The diagnosis has been confirmed by direct enzymatic assay in leukocytes. The deficient enzyme was N-acetyl- galactosamine-6-sulfate-sulfatase.

## Discussion

Acetabular damages have been described as a biomechanical cause of hip pain in active adult patients. Degeneration and alteration of the acetabulum in adults have been suggested as causes of hip pain and precursors to osteoarthritis of the hip. Damage to the acetabular labrum has been reported as a cause of irreducible dislocation or recurrent dislocation after traumatic dislocation of the hip. Four causes of acetabular damage have been proposed; trauma, hypermobility, bony impingements and dysplasia [5,6].

The mucopolysaccharoidoses (MPSs) share a chronic progressive course with multisystem involvement, several physical features, and radiographic abnormalities in conjunction with specific laboratory findings. Patients with Morquio syndrome usually can be clinically distinguished from patients with other forms of mucopolysaccharidoses. Morquio syndrome patients do not have the typical coarse facial features or mental retardation as seen in the other forms of MPSs. Their skeletal abnormalities are basically a spondyloepiphyseal dysplasia associated with ligamentous hyperlaxity [1-4].

Morquio syndrome is characterised by severe skeletal abnormalities with cloudy corneae and aortic regurgitation. Onset may be in the first two years of life with genu valgum, a short trunk and neck, pectus carinatum and coarse facies. Clouding of the cornea is mild but deafness may be a problem. The joints may be loose without the claw hand seen in other mucopolysaccharidoses. The Morquio syndromes belong to a family of disorders identified as lysosomal storage diseases, and historically as the mucopolysaccharidoses. These disorders are characterized by the lysosomal accumulation of glycoconjugates (glycolipids, glycoproteins and glycosaminoglycans) due to deficiencies in lysosomal hydrolases responsible for the degradation of these classes of molecules [7,8]

Previous reports described the variable clinical presentations in some patients with MPS type IV. Significant variation regarding the age at presentation as well as the chief complaint differed in each case. Fang-Kircher et al [9] described Perthes disease as the prime presentation in a patient with Morquio syndrome. Kanazawa et al [10] reported a man who began to experience hip pain after walking and standing erect for 30 minutes, which he was able to relieve by abducting his hip while standing. Radiographs revealed a central area of absorption in the main load-bearing area of the femoral heads, but no abnormalities in the other joints. Laboratory tests suggested the diagnosis of Morquio disease type IVA. Hecht et al [11] described the mild clinical manifestations in connection with Morquio disease.

In Morquio syndrome, both mortality and morbidity are related primarily to atlantoaxial subluxation resulting from the instability of odontoid process. A minor fall or excessive neck extension can result in cord transection and subsequent quadriparesis/death.

Although the skeletal changes may result in neurologic complications, but patients with the severe phenotype usually do not survive past the second or third decade of life. Odontoid hypoplasia leads to atlantoaxial instability. In recent years, surgical intervention of craniocervical fusion has been proposed to prevent this complication [12].

## Conclusion

Acetabular dysplasia can result in instability of the hip, joint incongruity, abductor insufficiency, and limb-length inequality. The condition is most commonly associated with a subtle abnormality of the hip joint at birth and or later on and in many occasions remains undetected for many years. Finally, we wish to stress that early detection of the underlying pathophysiology and radiographic assessment of the skeleton are major factors to delineate the diagnosis and to designate treatment.

## Abbreviations

SD: Standard deviation; MPS: mucopolysaccharoidosis.

## Consent

Written informed consent was obtained from the parents for the purpose of publication of the manuscript and figures of their child. A copy of the written consent is available for review by the editor-in-Chief of this journal.

## Competing interests

The authors declare that they have no competing interests.

## Authors' contributions

All of the authors were involved in the clinico-radiographic assessment and finalising the paper. All authors have red and approved the final version of the paper.

## References

[B1] Morquio L (1929). Sur une forme de dystrophie osseuse familiale. Arch Med Enf.

[B2] Brailsford JF (1929). Chondro-osteo-dystrophy. Am J Surg.

[B3] Beck M (1991). Mucopolysaccharidoses: nosology, clinical symptoms, therapeutic regimen (in German – English summary). Monats Kinderheilkd.

[B4] Beck M, Glossl J, Grubisic A, Spranger J (1986). Heterogeneity of Morquio disease. Clin Genet.

[B5] Klaue K, Durmin CW, Ganz R (1991). The acetabular rim syndrome. A clinical presentation of dysplasia of the hip. J bone Joint Surg Br.

[B6] Ranawat AS, Kelly BT (2005). Function of the labrum and management of labral pathology. Operative techniques in orthopaedics.

[B7] Fukuda S, Tomatsu S, Masue M (1992). Mucopolysaccharidosis type IVA. N-acetylgalactosamine-6-sulfate sulfatase exonic point mutations in classical Morquio and mild cases. J Clin Invest.

[B8] Laradi S, Tukel T, Khediri S (2006). Mucopolysaccharidosis type IV: N-Acetylgalactosamine-6-sulfatase mutations in Tunisian patients. Mol Genet Metab.

[B9] Fang-Kircher SG, Böck A, Fertschak W, Schwägerl W, Paschke E (1995). Morquio disease in a patient diagnosed as having Perthes disease for 38 years. J Inherit Metab Dis.

[B10] Kanazawa T, Yasunaga Y, Ikuta Y, Harada A, Kusaka O, Sukegawa K (2001). Femoral head dysplasia in Morquio disease type A: bilateral varus osteotomy of the femur. Acta Orthop Scand.

[B11] Hecht JT, Scott CI, Smith TK, Williams JC (1984). Mild manifestations of the Morquio syndrome. Am J Med Genet.

[B12] Ransford A, Crockard HA, Stevens JM, Modaghegh S (1996). Occipito-atlanto-axial fusion in Morquio-Brailsford syndrome. J Bone Joint Surg B.

